# An intervention to decrease burnout and increase retention of early career nurses: a mixed methods study of acceptability and feasibility

**DOI:** 10.1186/s12912-020-00524-9

**Published:** 2021-01-13

**Authors:** Judy Brook, Leanne M. Aitken, Julie-Ann MacLaren, Debra Salmon

**Affiliations:** School of Health Sciences, City, University of London, Northampton Square, London, EC1V 0HB UK

**Keywords:** Acceptability, Early career nurse, Feasibility, Intervention, Mixed methods, Nurse retention, Nurse workforce, Burnout

## Abstract

**Aims:**

To understand the experiences of nursing students and academic staff of an intervention to decrease burnout and increase retention of early career nurses, in order to identify acceptability and feasibility in a single centre.

**Background:**

Internationally, retention of nurses is a persistent challenge but there is a dearth of knowledge about the perspectives of stakeholders regarding the acceptability and feasibility of interventions to resolve the issue. This study reports an intervention comprising of mindfulness, psychological skills training and cognitive realignment to prepare participants for early careers as nurses.

**Methods:**

This is an explanatory sequential mixed methods study, conducted by a UK university and healthcare organisation. Participants were final year pre-registration nursing students (*n* = 74) and academics (*n* = 7) involved in the implementation of the intervention.

Pre and post measures of acceptability were taken using a questionnaire adapted from the Theoretical Framework of Acceptability. Wilcoxon Signed Ranks test was used to assess change in acceptability over time. Qualitative data from semi-structured interviews, focus groups and field notes were thematically analysed, adhering to COREQ guidelines. Data were collected February to December 2019.

**Results:**

One hundred and five questionnaires, 12 interviews with students and 2 focus groups engaging 7 academic staff were completed. The intervention was perceived as generally acceptable with significant positive increases in acceptability scores over time. Student nurses perceived the intervention equipped them with skills and experience that offered enduring personal benefit. Challenges related to the practice environment and academic assessment pressures. Reported benefits align with known protective factors against burnout and leaving the profession.

**Conclusion:**

Planning is needed to embed the intervention into curricula and maximise relationships with placement partners. Evaluating acceptability and feasibility offers new knowledge about the value of the intervention for increasing retention and decreasing burnout for early career nurses. Wider implementation is both feasible and recommended by participants.

## Background

The retention of nurses in the international healthcare workforce is a persistent issue, with nurse vacancies increasing in many high and middle-income countries. Nurses are integral to strong and resilient health systems but current deficits between supply, demand, and population need threaten to impact on universal health coverage goals [1]. Consideration of strategies to mitigate the nursing workforce deficit has become central to health policy nationally and globally [1, 2].

Increasing the number of student nurses as a pipeline to supply the workforce is a central approach to meeting need, and newly qualified nurses form the largest group entering the profession. However, these nurses are also particularly vulnerable [3], with 30–60% leaving their first place of employment within 1 year [4–6]. The transition from student to qualified nurse can be overwhelming, especially in a complex, fast-paced and pressured work environment [7]. This situation is compounded by rising nurse vacancies and has the potential to lead to burnout.

Burnout is a concept related to negative perception of the work environment, often linked with decisions to leave the nursing workforce [8]. It is characterised by depleted personal and/or social resources [9] and has significant consequences for healthcare organsiations, the individual and the patient population [8]. Known organisational and individual predictors of burnout allow interventions to be designed that will not only impact on burnout but also attrition from the profession.

This paper reports on the feasibility of implementation of an intervention to decrease burnout and increase retention of early career nurses. The perspectives of nursing students and university academic staff were sought in order to measure acceptability and identify barriers and facilitators to implementation and assess future scope for wider implementation.

Many examples of initiatives to support newly qualified nurses to stay in post and the profession have been reported. A large systematic review [10] identified key characteristics of interventions that were effective, with inconsistent or limited benefits frequently identified. The review exposed gaps related to three areas: evidence citing the explicit involvement of student or newly qualified nurses in the design of these interventions; the perspectives of the student or newly qualified nurses about the acceptability of the interventions; and the feasibility of the interventions from the perspectives of all stakeholders.

The expression ‘nurse retention’ is often used interchangeably with ‘turnover’ or ‘intention to leave’, and such confusion of terms perpetuates ambiguity and lack of understanding [11]. In this paper, the term ‘nurse retention’ describes a focus on decreasing attrition and minimising nurse turnover, to keep nurses in an organisation’s employment.

The concept of nurse retention consists of four attributes: motivation, intention, and individual decision; strategy and intervention; geographic context; and attachment to work [12]. To capture individual decision making, development of any interventions to support retention should incorporate the perspectives of those participating. Exploring acceptability supports understanding of the interactions, relationships and sociocultural contexts that influence perspectives of the intervention [13], and how this impacts feasibility and outcomes. It is likely to be interaction between characteristics of the individuals involved, the structure and workplace culture of the delivery environment, and characteristics of the intervention that act as facilitators or barriers to implementation [14]. Exploring these uncertainties in a feasibility study allows a larger study greater chance of success [15] and informs any required redesign [16].

The study aimed to understand the experiences of nursing students and academic staff who were involved in the implementation of an educational intervention, aimed at identifying and measuring acceptability, with a view to identifying barriers and facilitators to implementation and assessing future scope for the intervention.

## Methods

### Design

**Fig. 1:**
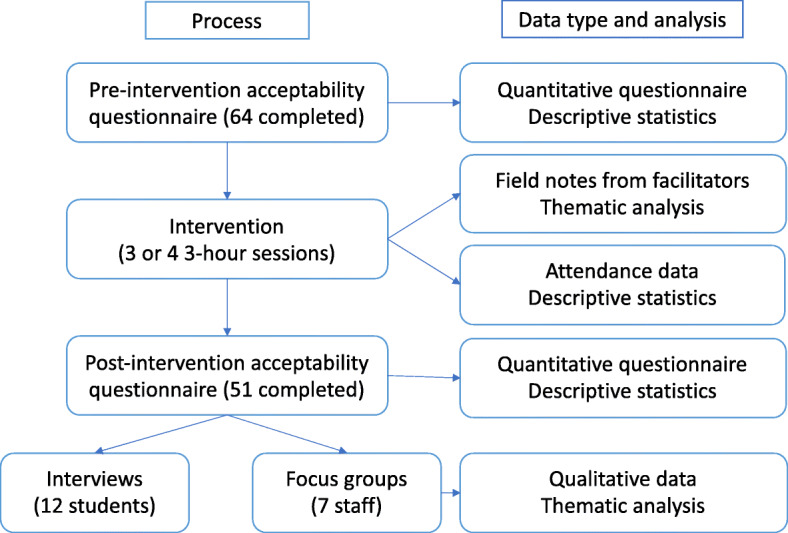
Diagram illustrating the mixed methods processes used in the study

This explanatory, sequential mixed methods research study combined insight from questionnaire data with participant accounts, providing a comprehensive evaluation of the feasibility and acceptability of the intervention. The sequence of processes is illustrated in Fig. 1.

This study was conducted as a partnership between a UK university and a large inner-city UK NHS healthcare organisation. The intervention was implemented at a university site familiar to the students. Intervention delivery and participant data collection occurred whilst the students were attending full-time clinical placements at the partnership NHS organisation sites, all situated within 10 miles of the University. The NHS organisation is located in a culturally and demographically diverse area, with the most unstable nursing workforce in England [17]. Relatively high cost of living, high population density, and proximity of many healthcare organisations in the city contribute to high turnover of nurses.

### Sample selection

Student participants in the study consisted of adult or child nursing students who were in the final year of their pre-registration nursing programme and who had engaged with the intervention. Academic participants included any members of the academic workforce who had been involved in the implementation of the intervention, including facilitators and nursing programme directors. All participants were purposively selected and invited by email and face-to-face to engage with the study.

#### Intervention

**Fig. 2:**
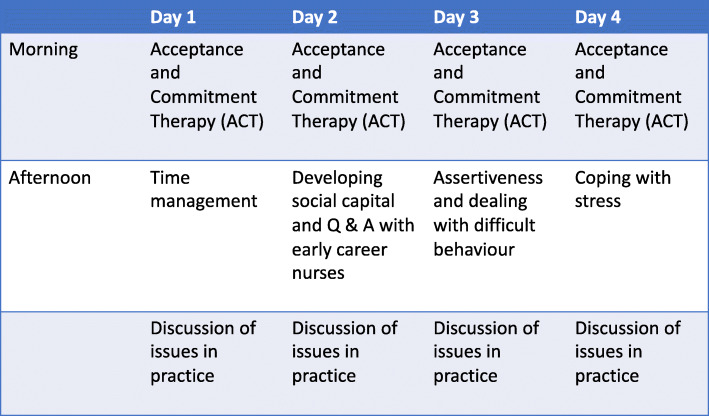
Content of the Intervention Activity Days

The intervention was co-designed by nursing students, early career nurses and researchers, drawing on evidence from the nursing literature, individual and collective experience. This innovative approach resulted in an intervention consisting of 3 or 4 days, called ‘Activity Days’, added to the nursing curriculum, in the final trimester of the pre-registration nursing programme between January and May 2019 for students working towards adult or children’s nursing qualifications. The content of the intervention is outlined in Fig. 2. Acceptance and Commitment Therapy is an evidence-based cognitive behavioural skills programme that helps people relate differently to difficult thoughts and emotions so they can construct their life around what really matters to them [18]. Social capital refers to the professional relationships, shared sense of identity, understanding, values and reciprocity that students and early career nurses develop with their colleagues to enable them to thrive in the workplace and is likely to have an impact on retention [19]. Sessions related to time management, assertiveness, coping with stress and opportunities to discuss any current issues with placements were also incorporated.

### Data collection

Quantitative questionnaire data on acceptability of the intervention and attendance data were collected, followed by qualitative data collection using semi-structured interviews, focus groups and reflective field notes. Data were collected between January and August 2019.

#### Attendance data

Student attendance at each of the intervention sessions was monitored and collated.

#### Questionnaire data

**Fig. 3:**
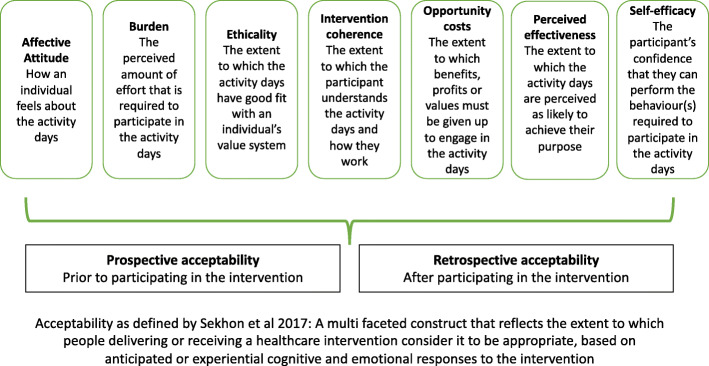
Theoretical Framework of Acceptability of Healthcare Interventions, adapted from Sekhon et al., [13], and applied to the intervention

Questionnaires based on the seven constructs of the Theoretical Framework of Acceptability of Healthcare Interventions [13] were developed (Fig. 3) and used a likert-type scale to collect data with additional free-text options. Open questions were included as a strategy to identify further issues for inclusion in the interviews and focus groups and to complement responses to closed questions [20]. The questionnaire detail is provided in Table 1.

**Table 1:** Detail of Acceptability Questionnaire Questions and scoring

Questionnaire: students were asked to circle the answer on a 5-point likert-type scale that best suited their opinion. Each question was phrased to reflect the temporal nature of the pre-and post-questionnaires.	
Question	Potential responses
1a. How acceptable do you feel it is to participate in these extra group and taught sessions? 1b. How acceptable do you feel it was to participate in these extra group and taught sessions?	Completely unacceptable (1); Unacceptable (2); No opinion (3); Acceptable (4) Completely Acceptable (5)
2a. I will enjoy taking part in the extra taught and group sessions. 2b. I enjoyed taking part in the extra taught and group sessions	Strongly disagree (1); Disagree (2); No opinion (3); Agree (4); Strongly agree (5)
3a. How much effort will it take for you to participate in the extra taught and group sessions? 3b. How much effort did it take for you to participate in the extra taught and group sessions?	Huge effort (1); Moderate effort (2); No opinion (3); Hardly any effort (4); No effort at all (5)
4. The extra sessions will be effective in helping me during my early career as a qualified nurse.	Strongly disagree (1) Disagree (2) No opinion (3) Agree (4) Strongly agree (5)
5a. I will value these extra sessions as a way of helping me get ready for my early career as a qualified nurse. 5b. I valued these extra sessions as a way of helping me get ready for my early career as a qualified nurse.	
6a. Participating in these extra sessions will interfere with my other priorities. 6b. Participating in these extra sessions interfered with my other priorities.	
7a. How confident do you feel about participating in the extra taught and group sessions? 7b. How confident did you feel about participating in the extra taught and group sessions?	Very underconfident (1) ; Underconfident (2); No opinion (3); Confident (4) Very confident (5)
8. It is clear to me how participating in the extra taught and group sessions will help me to cope better with the transition from student to qualified nurse.	Strongly disagree (1); Disagree (2); No opinion (3); Agree (4); Strongly agree (5)

A pretest-posttest quasi-experimental design was used to explore changes in participant perceptions of acceptability; questionnaires were completed prospectively (prior to experiencing the intervention) and retrospectively (after experiencing the intervention) to explore changes in perception of acceptability over time. Question phrasing was changed for each data collection point to reflect the temporal nature of the process.

#### Semi-structured interviews with students

Students who volunteered were interviewed, either face-to-face or by telephone and audio recorded. Each participant was only interviewed once. The topic areas were derived from the questionnaire data and included what went well, what could be changed, challenges, perceptions of relevance and benefit, and future scope of the intervention. Interviews were conducted using a conversational style that involved questions and prompts where appropriate. Interviews lasted on average 40 min and only involved the interveiwer and interviewee.

#### Staff focus groups

Academic staff were invited to take part in a focus group to discuss their experience. Topic areas were determined by issues raised in the questionnaire data and included logistical challenges, relevance and appropriateness of the intervention content, ideas for improvement and future scope, and any unintended consequences of the intervention on curricula or clinical placements. Focus groups involved only the participants and the facilitator, lasted 1 h and were audio recorded.

#### Reflective field notes

Intervention facilitators were invited to provide reflective notes about their experience of the sessions, particularly how they felt as facilitators, what went well, what was challenging and how the process could have been improved. Four of the 6 facilitators provided field notes.

### Ethical considerations

Ethical approval and permission to conduct the research was gained from the university and health service. Specific consideration was given to the voluntary nature of participation, including lack of coercion, the need for informed consent and respecting the anonymity of the participants. Participation was voluntary and informed consent was gained from all participants at the beginning of the study and repeated prior to participation in interviews or focus groups.

### Data analysis

Questionnaire data were analysed initially by all members of the research team to inform the discussion points for the interviews and focus groups.

#### Quantitative data

Acceptability questionnaire descriptive statistics were reported as frequency of response choice for each question. Wilcoxon signed ranks were used to compare pre and post intervention paired mean ranks to assess difference over time. Attendance data were reported as frequency for each session and each student group.

#### Qualitative data

Thematic analysis of acceptability questionnaire free text comments, focus groups, interviews, and field notes followed Braun and Clarke’s [21] six phases. Data from staff and student participants were analysed separately. Data analysis was supported by use of Nvivo software V12.

### Validity and reliability/rigour

The multi-component Theoretical Framework of Acceptability supports an acceptability issue to be identified at source and allows refinement of an intervention prior to wider implementation [13]. To enhance content and construct vaidity, the questionnaire was piloted with a small group of non-participants and reviewed by the framework author, after which small changes to wording were made to improve clarity. Questionnaires were self-administered in the presence of the intervention facilitators. Use of participant numbers mitigated social desirability bias by reassuring participants of the confidentiality of their answers.

Interview and focus group guides were formulated following analysis of questionnaire data and decisions about which aspects required further explanation. Interviews with students were conducted by two female researchers; a nurse with experience and training in qualitative research, and an experienced academic with a PhD in Psychology. The researchers facilitated the intervention but did not have any previous relationship or ongoing influence. Focus groups with academic staff were conducted by an experienced female academic post-doctorate researcher independent to the staff group, with no prior relationship, ongoing influence or previous nursing experience. The researchers introduced themselves and the study to the participants at the start of the focus groups and interviews. For the duration of the study the research team met regularly for reflexive discussions to explore their biases, assumptions and relationship to the research topic. All qualitative data were collected on University premises or by telephone. Data collection ceased once all volunteers had been interviewed, recurring themes were noted by the interviewers and data saturation was deemed to have been reached.

To preserve the anonymity of the participants, and mitigate bias, each participant was assigned a number and this was thereafter used to identify quotes from interviews or field notes.

## Results

Seventy-four students engaged with the intervention and 70 completed the acceptability questionnaire at one or more time points. The mean age of student participants who attended 1 or more intervention sessions and completed 1 or more acceptability questionnaires was 26 years (SD 6.54). Fifty (71%) were undertaking adult nursing programmes and 20 (29%) undertaking child nursing programmes, with 47 (67%) studying for a bachelor’s degree (BSc) and 23 (33%) studying for a post graduate diploma (PGdip). Twelve students were interviewed, and 7 academic staff attended one of two focus groups. Academic staff were either Lecturers or Senior Lecturers teaching on the nursing programmes. As participants were volunteers, reasons for non-participation in the interviews or focus groups are unknown.

### Attendance

Few students attended all of the intervention sessions, however, highest attendance (72–84%) was for the mixed group of adult and child nursing post graduate diploma students. As this was the second degree for these students, they were older, more experienced at studying and potentially more experienced at managing multiple priorities. Lowest attendance was for BSc undergraduate degree child nursing students (26–41%). These students were the youngest group, the first cohort to receive the intervention and were working full-time in their final placement, which was critical to complete their academic programme to qualify as nurses. Further detail is given in Table 2.

**Table 2:** Attendance by session and degree

		Number (%) attended by session^a^				
Degree	No. invited to attend (baseline)	Session 1	Session 2	Session 3	Session 4	Attended 2 or more sessions
**BSc Adult**	47	26 (55%)	28 (60%)	28 (60%)	24 (51%)	37 (79%)
**BSc Child**	27	7 (26%)	11 (41%)	11 (41%)	8 (30%)	14 (52%)
**PGDip Adult & Child**	25	21 (84%)	19 (76%)	18 (72%)	N/A	23 (92%)

^a^For PGdip students, the same content was compressed into three sessions due to timetabling restraints

### Acceptability questionnaire results

One hundred and five questionnaires were completed (64 pre and 51 post). Students found the intervention generally acceptable and their perception of acceptability increased pre to post intervention (Table 3). For five of the seven acceptability constructs, a significant positive increase in perception occurred. Students enjoyed the intervention more over time, increasingly perceived that it was effective, felt the intervention fitted with their personal values, gained clarity and understanding about the intervention, and became more confident in their ability to take part. Conversely, responses to questions related to the extent to which taking part interfered with other priorities, indicated that over time the opportunity cost of participating became significantly greater. Perceived effort to participate, relating to the construct of burden, trended towards an increase over time but not significantly. For most items on the acceptability questionnaire there was no association between perceptions of acceptability prior to participation and frequency of attendance, although participants who attended 2–4 sessions (compared to 1 session) were more likely to agree that they were clear how participating would help them to cope better with the transition from student to qualified nurse (χ2 13.53, *p* = 0.004) (Table 3).

**Table 3:** Intervention acceptability pre- and post intervention

	Median (Interquartile range)		Wilcoxon Signed Ranks test	
Question number/construct	Pre-intervention	Post-intervention	Z	***p***
1. Overall acceptability	4 (4–5)	5 (4–5)	−2.78	0.006**
2. Affective attitude	4 (3–4)	5 (4–5)	− 4.06	< 0.001***
3. Burden	2 (2–4)	2 (2–4)	−1.82	0.066
4. Perceived effectiveness	4 (3–4)	5 (4–5)	−3.60	< 0.001***
5. Ethicality	4 (3–4)	5 (4–5)	−2.58	0.009**
6. Opportunity costs	3 (2–4)	4 (2–4)	−2.91	0.003**
7. Self-efficacy	4 (3–4)	4 (4–5)	−2.1	0.040*
8. Intervention coherence	4 (3–4)	5 (4–5)	−3.92	< 0.001***

Key: * < 0.05, ** < 0.01, *** < 0.001

### Qualitative findings

**Fig. 4:**
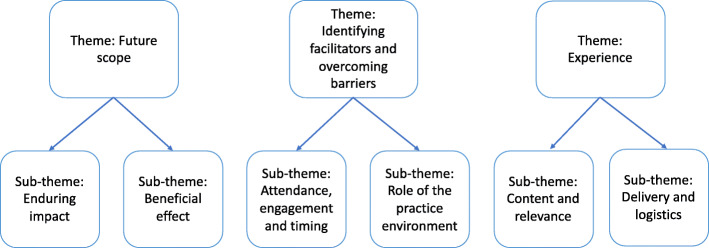
Themes and sub-themes derived from qualitative data

Thematic analysis conducted on interview and focus group data, reflective field notes and free text comments from the acceptability questionnaires, identified three themes in both student and academic staff data: experience; identifying facilitators and overcoming barriers; and future scope. Six subthemes included: content and relevance; delivery and logistics; attendance, engagement and timing; role of the practice environment; enduring impact; beneficial effect (Fig. 4).

### Experience

The experience of participating in the intervention was generally reported positively by both students and staff, reiterating findings from the student acceptability questionnaire that affective attitude was more positive over time. Students predominantly commented on content of the intervention and staff commented on facilitation.

#### Content and relevance

Most students felt the content was appropriate and relevant to their roles as student and qualified nurses, perceiving the subject matter to be a positive addition to the traditional curricula. The focus on clinical practice and skills to support transition to early career nurse was particularly welcomed.

*It was everything that I felt like University hadn’t identified as important, which was actually so important in practice, you incorporated it into a three-week course. (student 9 interview)*

These sentiments were echoed by the staff, who identified that the co-produced nature of the intervention lent additional credibility to the content. As highlighted in the questionnaire data related to intervention coherence, students understood the rationale behind the intervention and recognised the relevance of the content of the sessions.

#### Delivery and logistics

The nature of the intervention, incorporating psychological skills training, and small and large group work, encouraged facilitators to limit the sense of hierarchy in the groups, by appropriately sharing personal and work experiences. The students commented positively about the helpful and respectful approach of the facilitators, which made sharing difficult experiences in placement possible.

*It just gives you time to relate to everyone else, because everyone spoke about their experiences and no one was judging anyone, everyone was just saying you know what, this is what happened in my placement...no one’s there to put you down. (student 1 interview)*

The compassionate facilitation of the sessions aligns with the self-efficacy scores in the acceptability questionnaire, with students becoming increasingly confident over time that they could contribute effectively.

### Identifying facilitators and overcoming barriers

Across the data, barriers and facilitators to implementation of the intervention were highlighted. Staff discussed their learning from working in partnership with a large healthcare organisation and acknowledged the complexity of trying to retrofit additional sessions into established curricula. Students discussed conflicting priorities, lack of initial confidence as they found their way in new clinical placements, and the influential nature of the clinical environment on their decision making.

#### Attendance, engagement and timing

Engagement with the intervention was varied. In the last trimester of the programme students had many priorities, including academic and clinical practice assessment deadlines, and full-time placement responsibilities. Staff recognised the pressures the students were under and how this affected their decision to attend.

*As the pressure builds on a student through the third year … I think so does their ability to take on new things reduce because they’ve got so much going on like dissertations, final placements, clinical assessments and other coursework, things like that (staff FG 2)*

Some students identified that the days provided respite from the intensity of clinical practice and the sharing of placement experience was supportive and relevant. Others felt that attendance would have been much greater when the pressures of the programme were fewer.

*The problem is when we’re on placement we’re doing full time hours plus extra study sessions and then a lot of people have to work on top of that and then that doesn’t even include the people that have children and family … I find managing my life and prioritising things so challenging. (student 9 interview)*

The perception of increased burden or effort to attend over time indicated by the acceptability questionnaires reflects the conflicting priorities and pressures of the final stage of the nursing programme.

#### Role of the practice environment

Both students and staff commented about how clinical colleagues influenced their experience. Staff found that communication across the two organisations was challenging, with misunderstanding about the nature and value of the intervention. They recognised that the busy nature of the clinical areas was influential, as patient care was central to decision making but noted that clinical staff were sometimes reluctant to release students to attend. This impacted on student motivation to negotiate attendance at the sessions.

*It’s difficult because it’s supposed to be a collaboration between the [NHS organisation] and the University and the students seem to have been caught a little in the crossfire, but in most cases I think the students were able to get to attend when they needed to. (staff FG 2)*

*The placement, they don't like it, yeah, because it was final year placement … They keep telling us, “Well that's irrelevant ... you shouldn't be attending those. It's more important that you attend your clinical hours.” (student 6 interview)*

Many of the students described their clinical experience as stressful. The intervention was therefore timely as it stimulated a realisation that the stress needed to be addressed and provided new skills and tools to support transition to a new role. Students described how they had incorporated the new techniques into their everyday practice and were seeing positive results.

*The mindfulness I did find quite useful with the breathing, because the neuro placement was really hectic … I would just focus on myself and take five seconds, ten seconds to breathe and then I’ll be like OK, I’ve got to do this, this and this, and it helped me organise my head in a way. (Student 5 interview)*

The narrative from both students and staff describing clinical practice as a pressurised environment relates to the acceptability questionnaire data showing an increase in perceived burden and significant increase in opportunity cost for students participating in the intervention.

### Future scope

Both students and staff were unanimous in recommending that the intervention should be offered to all future students. Participants suggested that it should be introduced at the beginning of the nursing programme and continue as a fundamental aspect of learning until qualification. Both staff and students would encourage colleagues and peers to become involved with delivery and attendance. Staff strongly believed that the co-designed nature of the intervention gave it credibility as a response to the expressed needs of a changing demographic of students.

#### Beneficial effect

Students described many aspects of the intervention that had immediate benefit for them in both their professional and personal lives. They felt the sessions gave them insight into transition to a qualified nurse but also helped with their placement experiences as a student. Of particular benefit were the mindfulness and psychological skills and techniques, which students felt helped them to cope better with their emotions in difficult situations. Likewise, the opportunity to meet with peers in a safe environment to discuss placement experiences and strengthen networks was highly valued.

*It was nice that no one thought we were negative people when we discussed negative things. Most people say we should just be positive but you taught us how to and that its okay to struggle slightly. (Child BSc Student Acceptability Questionnaire 11)*

For some students the intervention was a lifeline at a difficult period of their lives.

*I genuinely appreciated those sessions, as a student, and in terms of personal life as well. At one point it got quite emotional for me, because I thought, my goodness, this is really helpful. And, finally there’s a focus on us students, and our mental wellbeing. (student 7 interview)*

Both students and staff highlighted personal benefits of the intervention and crucially, students recognised the need to be aware of their own wellbeing, notice how they were responding to stress and take proactive action.

*You get the stress where you just brush it aside but it affects us, but the sessions made me realise it affects us a lot more than we think and that if we didn’t deal with it, it has such bad effects and I think that was the, the sessions helped me come to a realisation. (student 1 interview)*

#### Enduring impact

Students described activity connected to time management, stress management and coping mechanisms related to both in their work and home lives. Although not all students were consistently using specific skills there was a sense that the sessions had changed their perspective and they could draw on the techniques and newly developed networks at difficult times.

*I felt that the sessions helped me to cope with the stress and I have taken away skills that I can apply not just within nursing but in everyday life. (Adult BSc Student Acceptability Questionnaire 13)*

Students described embedding their learning into their daily activities, for example by downloading mindfulness apps onto their phones and practising meditation or breathing exercises during their commute or at break times on the wards. Some described how they now accepted negative thoughts and were more conscious of how their behaviour could reflect their personal values and impact on colleagues, friends and family; key tenets of ACT.

*Meditating I tried, but it's just so difficult when you're stressed … it's good because it made me aware of, OK my heart's racing, OK I, you know, I can't seem to breathe properly. It was great to be aware and notice your feelings, but I wasn't able to put myself in the full meditation mode. (student 6 interview)*

Staff also recognised the benefits for students and felt that the intervention would have enduring impact. They noticed subtle changes in the students’ thinking and demeanour. The staff members anticipated that the new skills would support students to deal with the challenges, such as low self-esteem, helplessness and home-life responsibilities.

*What they were doing as part of this study actually began to bleed through into some of their thinking about other things, which is difficult to capture objectively but … I think it was a really positive experience (staff FG 1)*

Staff regarded the intervention as positive enhancement of the traditional university offer that would be beneficial to the students in their future career. This positive perception of the immediate and enduring impact of the intervention by both students and staff aligns with the acceptability questionnaire data indicating a significant increase in perception of the intervention as an effective mechanism for supporting early career nurses.

## Discussion

The deficit of nurses in the healthcare workforce has motivated a plethora of initiatives to encourage retention of newly qualified nurses but to date there is little published research about the acceptability of these interventions [10]. To understand more about the impact of the intervention, this study was designed to explore the experiences and perceptions of nursing students and academic staff during implementation of a novel intervention. The findings raised key points for discussion: first, the intervention was generally acceptable and scores for five of the seven specific acceptability constructs showed positive significant increases over time, indicating a sound platform for wider implementation. Second, the intervention was perceived by student nurses to equip them with skills and experience that brought enduring personal benefit in both their professional and personal lives, with the potential to influence their transition to qualified nurses, burnout and retention. Third, implementation of the intervention in partnership with an NHS organisation and within established higher education curricula added a level of complexity that influenced student attendance and feasibility.

Acceptability is increasingly being recognised as a key aspect of developing healthcare interventions [22]. Strengths of the Theoretical Framework of Acceptability [13] include the definition, the multi-faceted construct, and prospective and retrospective assessment of acceptability. Individuals may perceive an intervention differently before they experience it than after.

Students did indeed significantly change their perception of intervention acceptability over time, reinforcing the value of temporal assessment to support decisions about feasibility. For five of the seven constructs this was a positive change as they gained clarity, understanding and expertise, highlighting how clear and timely explanations about the intervention may have engendered greater participation.

Findings for two acceptability constructs indicated a less positive trend in perception of the intervention. Over time the perceived cost of participating became greater, as did the perceived effort to participate. Data from the interviews and focus groups indicated a potential relationship between the timing of the delivery of the intervention in the nursing programme and other academic and practice related priorities. This was evident in the language students used in their interviews, frequently referring to stress and pressure, compounded by accounts of busy and under-staffed placement environments. Stress related to nursing programmes is a recognised phenomenon [23], is associated with academic, clinical or personal/social stressors [24] and there is a strong relationship between stress and attrition [25, 26]. In particular, students experience moderate to severe levels of stress during clinical practice [27], highlighting the importance of addressing both individual stress and organisational stressors [28] to mitigate the impact on students. Wider implementation would require careful planning to manage the complexity of accommodating the intervention in an established nursing programme, avoid work overload and placement anxiety and limit the impact on attendance.

Perceptions of burden and opportunity cost were also influenced by the relationship with the NHS organisation. The findings indicate that the participants were cognisant of the power difference between students and supervisors, assessors, and ward managers in the practice areas. Relationships with clinical staff are frequently cited as a stressor for nursing students [23], with effective supervision and a supportive environment critical to students feeling a sense of empowerment [29]. Where learning is not optimised, competence and confidence can be affected [30, 31] and this may explain the difficulty students reported when deciding to engage with the intervention. For wider implementation preparatory work is essential to embed understanding about the value of the intervention and facilitate prioritisation. Consideration should be given to both student and NHS colleagues’ commitments to maximise engagement and work in partnership. Future work around feasibility should include the views of a wider range of stakeholders from the NHS organisation. Despite perceptions of increased burden and opportunity cost, all those interviewed were unanimous in recognising the future potential of the intervention to support student and early career nurses.

A key facilitator to wider implementation was the innovative nature of the content of the intervention, which drew on theories of stress and wellbeing. The effective combination of exposure to new knowledge, together with a cognitive reappraisal and relaxation techniques [28], was enhanced by additional exposure to new knowledge about early career nursing practice. Students reported enduring personal benefits at the post intervention interviews, which will have influenced perceptions of acceptability and motivated attendance but also have the potential to mitigate burnout. Strategies to enhance personal wellbeing may help to support newly qualified nurses to cope in an environment with high emotional demand, understaffing or challenges with communication [32]. If a nurse feels they are upholding their personal values in their professional work, it may offer psychological protection and increase satisfaction with the workplace [33]. Similarly, such personal benefits may impact on nurse retention by increasing motivation, perceptions of job control and individual decision making, and attachment to the profession and the organisation [12].

### Strengths and limitations

The strength of this study lies in the novel approach to development and application of the intervention, spanning boundaries between nursing practice and higher education. To our knowledge, the intervention is of unique design and the findings are relevant to a range of both academic and healthcare settings. The mixed methods design, the range of participant voices and the breadth of data sources further strengthens the credibility of the acceptability and feasibility, with qualitative data adding depth and contextualisation to questionnaire data.

A potential limitation is the attendance at the intervention. Although 70 acceptability questionnaires were completed, few students attended all intervention sessions and little is known about the views of those who did nto attend. However, the qualitative data helped to contextualise the lower attendance. The single study site is a further limitation. Additional studies of feasibility are recommended across an increased number of sites with different characteristics.

## Conclusion

Nurse retention is a global concern and focusing on preparing student nurses for their early career in order to decrease burnout and increase retention is an essential element of strategies to address the issue. This study was designed in recognition of the importance of intervention acceptability to successful implementation and offers a novel approach that explores both prospective and retrospective participant perceptions. The findings indicate that not only was the intervention perceived as acceptable but the positive perception increased over time. Although challenges of the practice environment and pressures of academic assessment impacted on attendance, the personal benefits reported by participants align with known protective factors against burnout and decisions to leave the profession. Wider implementation would require careful planning to incorporate the intervention into curricula and maximise the potential of the relationship with practice partners. Evaluating acceptability and feasibility of the intervention offers new knowledge about the value of the content and allows us to conclude that wider implementation is both recommended by participants and feasible.

## Data Availability

The datasets generated and/or analysed during the current study are not publicly available due to the need to maintain the anonymity of participants but are available from the corresponding author on reasonable request.
